# The spatial distribution of the U.S. childfree population: Does state context matter?

**DOI:** 10.1371/journal.pone.0352872

**Published:** 2026-08-12

**Authors:** Zachary P. Neal, Jennifer Watling Neal

**Affiliations:** Psychology Department, Michigan State University, East Lansing, Michigan United States of America; University of Salamanca, SPAIN

## Abstract

Childfree adults do not have and do not want children, and represent a large and growing fraction of the total population in the United States. However, we know relatively little about where childfree adults live, or whether the features of their state’s context are associated with being childfree. In this study, we use data from 20,118 respondents in all 50 states to better understand the childfree population’s spatial characteristics. Despite substantial between-state variation in prevalence, from 22% in California to 9.4% in Louisiana, there is no evidence that this population is clustered in specific multi-state regions. Additionally, mixed-effects model estimates suggest that although being childfree was more common in more populated states with higher costs of living, individual demographic characteristics are much more strongly associated with being childfree. We conclude by discussing the policy implications of these findings, and identifying future directions that are highlighted by this study’s limitations.

## Introduction

Childfree adults do not have children and do not want to have children in the future. They represent a large and growing fraction of the total population in the United States (U.S.) [1,2], with nearly 30% of all non-parents identifying as childfree in 2023 [3]. The childfree population is important not only due to its size and growth, but also because it has implications for the country’s long-term population growth and because its members often have unique health, work, and financial needs [4–6]. However, despite this population’s importance, we know relatively little about where childfree adults live, or whether the features of their state’s context are associated with being childfree.

In this study, we used data from each U.S. state to better understand this population’s spatial characteristics. First, we mapped the prevalence of childfree adults in each state, finding that despite substantial between-state variation (e.g., 22% in California, 9.4% in Louisiana), there was no evidence that this population is spatially clustered in specific multi-state regions. Second, we examined state characteristics that were associated with being childfree, finding that being childfree was more common in more populated states with higher costs of living. Finally, we compared the association of being childfree with both individual and state characteristics, finding that being childfree was much more closely associated with individual demographic characteristics than with state contextual characteristics.

This paper is organized in four sections. First, in the background section, we define the childfree population and review prior research on its prevalence, spatial distribution, and correlates. Second, in the methods section, we introduce the Civic Health and Institutions Project, a 50 States Survey’ (CHIP50) data, describe how we identify respondents who are childfree, and explain the analyses we use to answer each research question. Third, in the results section, we report findings from the analyses designed to map the spatial distribution of the U.S. childfree population and identify individual and state characteristics associated with being childfree. Finally, we conclude in the discussion section by considering the policy implications of our findings, and identifying future directions that are highlighted by this study’s limitations.

## Background

Fertility research focuses on when or whether people have children, and thus focuses on behaviors. However, when studying non-parents (i.e., people who have not had children), it is also possible to focus on their attitudes, and specifically whether they want children. An individual is ‘childfree’ when they do not have biological or non-biological children, and do not want children in the future [7]. This distinguishes childfree individuals from several other types of non-parents, including ‘childless’ individuals who cannot have children but wanted them, ‘not yet parents’ who want to have children in the future, and ‘undecided’ individuals who are unsure whether they want children.

We adopt the definition of childfree proposed in a recent framework for research on this population [8], which has two unique features. First, this framework defines being childfree as a ‘momentary status’ that may change over time. For example, a childfree individual may decide to have children and thus become a parent (childfree → parent), or an undecided person may decide they do not want children and thus become childfree (undecided → childfree). Second, this framework also defines childfree solely in terms of desire, and not in terms of fecundity. Therefore, a person who is infecund (i.e., who cannot have children) may still be childfree if they do not want children anyway.

This study’s focus on the childfree population imposes a few scope conditions that are important to acknowledge at the outset. First, we focus on individuals who are *currently* childfree using cross-sectional data. Examining the spatial distribution of the childfree population from a life course perspective that considered whether childfree individuals remain childfree (or non-childfree individuals become childfree) is also interesting, but would require spatially-explicit panel data that does not exist, and thus is beyond the scope of this study. Second, we focus on individuals who do not want to have children, regardless of whether they are able to have children. Thus, we focus on individuals’ (lack of) desire for children, and not on their fertility or fecundity, which are biological phenomena that lie beyond the scope of this study. Finally, although there are many types of non-parents, we focus only on childfree non-parents because they are substantially more common than childless non-parents, and because they intend to remain non-parents [3,9]. The spatial distribution of rare types of non-parents (e.g., childless) or of non-parents who plan to become parents is beyond the scope of this study, but in principle could be explored using the data we share.

The childfree population is important to understand for several reasons. First, childfree individuals have potentially unique needs that differ from parents or other types of non-parents, such as access to pharmaceutical or surgical birth control [5], financial planning that does not involve dependents or heirs, and preparation for end-of-life care [6]. Second, because having (and wanting to have) children is normative, childfree individuals are often stigmatized [10–12], and may be subject to additional expectations in the workplace that impede work-life balance [4]. Finally, the childfree population plays an important role in fertility rate and population growth trends, and thus must be considered to obtain accurate population forecasts.

Estimating the size of the U.S. childfree population is challenging because nationally representative demographic data often do not include the information necessary to determine which respondents are childfree. Studies that do provide estimates often only focus on selected states (e.g., Michigan) [13] or subgroups (e.g., older women) [14]. However, recent estimates from the National Survey of Family Growth suggest that the prevalence of childfree individuals in the U.S. is large and has grown over the past two decades, from 13.8% of non-parents ages 15–44 in 2002 to 29.4% in 2023 [3].

### Spatial distribution of childfree people

Despite the increasing size of U.S. childfree population, relatively little is known about where this population is located. Some research suggests that there may be significant state-level variation in the prevalence of childfree people. For example, childfree individuals were substantially more common in Michigan [15] than nationwide in 2022 [3]. One common expectation is that childfree individuals are drawn to cities for access to amenities, and are not deterred by smaller urban houses or larger urban schools. However, findings about the relationship between urbanicity and being childfree have been mixed. Although one study found that childfree individuals were more likely to live in an urban area compared to parents [16], a review of nine other studies found no evidence that childfree individuals were more likely to live in an urban area [17].

Understanding the spatial distribution of the childfree population is important for several reasons. First, accurate population forecasts require considering regional variations in fertility intentions. For example, a state with a large and growing childfree population may follow a different growth trajectory than a state with a small and stable childfree population. Second, meeting the health and other needs of the population requires understanding local variations in those needs. For example, a state with a large childfree population may benefit more from clinics that provide birth control services than those that provide fertility services. Therefore, in this study we ask: *How is the U.S. childfree population spatially distributed, and is this population concentrated in certain areas* (**Research Question #1**)?

### The role of state context

Past research has examined a potential bidirectional effect between local context and whether or when people have children (i.e., fertility). Specifically, researchers have examined whether a person’s decision to have children affects where they choose to live (i.e., fertility → context) [18], and whether where a person lives [19–22] or grew up [23] affects whether they choose to have children (i.e., context → fertility). Although this work offers insight into the association between local context and whether people *do have children*, questions remain about the role of local context in whether people *want to have children*, and specifically whether people are childfree.

Although ‘local context’ can be examined at a variety of spatial levels, in this study, we focus on the state level because it is the governmental level at which potentially influential policy (e.g., on education, health access) is set in the U.S. To better understand how state context may be relevant to a lack of desire for children and the choice to be childfree, we explore four state-level characteristics. The choice of characteristics was informed by past fertility and childfree research cited below. It was also informed by a desire to focus on genuinely contextual features, as opposed to variables that are merely aggregations of individual-level characteristics (e.g., percent White) that can be more precisely modeled at the individual level (e.g., race). However, given the lack of prior cross-context or spatially-explicit research on childfree people in the U.S., this analysis remains exploratory and we do not offer formal hypotheses.

First, fertility research has found that non-parenthood is associated with local population density [20]. Measuring population density at the state level can be misleading because many states have significant rural and urban variation (e.g., New York City vs. the rest of New York state). However, total state population may also be associated with being childfree. For a given prevalence rate, states with larger populations will contain larger numbers of childfree individuals. These larger numbers of childfree individuals in the same state may not only offer community and support to existing childfree people, but may also lead others to identify as childfree by normalizing the choice not to have children.

Second, fertility research has examined the association between non-parenthood and a broad range of local economic conditions including neighborhood disadvantage [19], neighborhood social status [23], and gross domestic product [20]. Although these are not relevant to, or not computed at, a state level, they do suggest that economic considerations are important. Among the most salient economic conditions is the cost of living, which may be associated with being childfree. Research in the U.S. [24] and globally [25] has consistently found that the costs of raising children, and other economic concerns, are among the most commonly cited reasons for not having children. For example, in 2024, 36% of U.S. non-parents reported that their inability to “afford to raise a child” was among the major reasons they were unlikely to have children in the future.

Third, state abortion laws may be associated with being childfree because they shape the local risks involved in being pregnant or needing to obtain reproductive health care. Following the U.S. Supreme Court’s ruling in *Dobbs v. Jackson*, which overturned *Roe v. Wade* and allowed states to enforce their own restrictions on abortion, the number of people identifying as childfree [8] and pursuing voluntary sterilization [26] increased in states with fewer protections.

Finally, state support for public education may be associated with being childfree. There is limited prior research to support this possibility. However, we consider it because spending on public education is among the more visible ways that the state can invest in children, and signal support for parents and parenting. It is also more directly controlled by state policy than the other three characteristics, and thus if associated with being childfree, would have more direct policy implications.

Focusing on these four state contextual characteristics, we ask two related research questions. First, we ask: *Are a state’s population, cost of living, abortion laws, or support for education associated with being childfree* (**Research Question #2**)? Answering this question is important for understanding how broader contextual characteristics may shape the composition of a state’s population.

Second, we ask: *Are individual-level demographic characteristics or state-level contextual characteristics more closely associated with being childfree* (**Research Question #3**)? Past research has investigated whether being childfree is associated with an individual’s sex, marital status, age, race, political ideology, education, and the urbanicity of their residence [8,13,15–17]. Some fertility research has found that local context has a limited impact on fertility behaviors compared to these individual characteristics [19,22,23]. However, the relative strength of association of these two levels is unknown for attitudes about wanting children. Answering this question is important for understanding the potential effectiveness of state policy intended to encourage residents to have children by, for example, lowering the cost of living or boosting funding for education.

## Methods

### Data

To explore these research questions, we use data from the ‘Civic Health and Institutions Project, a 50 States Survey’ (CHIP50, https://www.chip50.org/). The CHIP50 is a multi-university collaborative effort to collect data from non-probability quota samples that are weighted to be representative of the U.S. as a whole, and of each state, thereby permitting inferences at multiple geographic levels. It is the successor to the earlier ‘COVID States Project,’ which began collecting data in April 2020 to examine COVID-related health and policy. Each wave of the survey now includes core demographic questions accompanied by a unique set of researcher-generated questions in multiple fields including political science [27], public health [28] and mental health [29].

In this study, we use data from the 33^rd^ wave of CHIP50, which was collected by the CHIP50 team between 30 August 2024 and 8 October 2024 via a web-based survey of U.S. adults, and which included questions that enables the identification of childfree adults [30]. To obtain state-level representative samples, they recruited quota samples from online respondent panels, collected data using the PureSpectrum online polling platform, then computed state-specific post-stratification weights with respect to race, ethnicity, age, gender, education, and geographic region. These data were cleaned in two stages. First, prior to releasing data to researchers, the CHIP50 team filtered bots using a CAPTCHA test, excluded respondents who failed attention checks, and excluded respondents who exhibited anomalous response or non-response patterns. Second, exploiting the additional information available in social network questions that were included in this wave, these data were further cleaned to exclude additional respondents who were suspected bots or who failed attention checks [31].

These cleaned data were accessed on 15 July 2025, and include 20,901 respondents. In the analyses reported below, we also exclude respondents living in Washington, D.C. (*N* = 327), respondents who failed to report a political ideology (*N* = 93), and respondents whose childfree status could not be assessed due to missingness on key variables (*N* = 366). Thus, we focus on a cleaned analytic sample of 20,118 respondents across 50 states. The Michigan State University Institutional Review Board determined that analysis of these data does not constitute human subjects research because the data are anonymous and publicly available (STUDY00009134).

## Variables

### Childfree status

The CHIP50 survey followed an existing measurement framework [7] for measuring respondents’ family status. First, all respondents were asked “Do you have, or have you ever had, any biological or adopted children?” and could respond yes, no, don’t know, or prefer not to answer. Second, respondents who answered ‘no’ to the first question were asked “Do you plan to have any biological or adopted children in the future?” and again could respond yes, no, don’t know, or prefer not to answer. Finally, respondents who answered ‘no’ to the second question were asked “Do you wish you had, or could have, biological or adopted children?” and again could respond yes, no, don’t know, or prefer not to answer. Respondents who answered ‘no’ to all three questions were classified as childfree, while respondents who answered ‘yes’ or ‘don’t know’ to *any* question were classified as not childfree. In this study, respondents’ childfree status was measured using a binary indicator variable equal to 1 for childfree respondents, and 0 for respondents who are not childfree.

### Individual characteristics

Respondent sex, race, and marital status were measured using binary indicator variables equal to 1 for male, White, and single respondents, respectively. Age was measured in years at the time of the survey. Political ideology was measured on a 7-point scale ranging from 1 for ‘extremely conservative’ to 7 for ‘extremely liberal.’ Education was measured on a 5-point scale ranging from 1 for ‘some high school or less’ to 5 for ‘graduate degree.’ Finally, the urbanicity of the respondent’s residence was measured using the 6-point U.S. National Center for Health Statistics’ urban-rural classification, which ranges from 1 for ‘non-core’ to 6 for ‘large central metro’.

### State characteristics

We focused on four state characteristics that can be measured directly at the state level, rather than by aggregating individual data within a state, and that theory and prior research suggested may be associated with being childfree. First, we measured state population using the logarithm of population in 2024 obtained from the U.S. Census [32]. Second, we measured cost of living using regional price parities (RPPs) in 2023 computed by the U.S. Bureau of Economic Analysis [33]; RPPs express the cost of living in a state as a percentage of the national average. Third, we measured protection for abortion rights on a 7-point scale ranging from 1 for ‘most restrictive’ to 7 for ‘most protective’ that was compiled by the Guttmacher Institute [34]. Finally, we measured support for public education using the logarithm of per pupil spending in 2020–2021 compiled by the U.S. National Center for Education Statistics [35].

### Analysis plan

Before using these data to answer the research questions, we first validated the data by estimating the prevalence of childfree adults nationwide and in Michigan, then by comparing these estimates to previously published estimates. Importantly, computing a national prevalence estimate from these data requires using the weights stored in the weight variable to ensure national representativeness, while computing state-specific prevalence estimates from these data requires using the weights stored in the weight_state variable to ensure state representativeness.

To answer Research Question 1 – *How is the U.S. childfree population spatially distributed, and is this population concentrated in certain areas* – we first computed the percent of each state’s population that was childfree using state-specific post-stratification survey weights. We then computed Moran’s I, where spatial proximity is defined by a shared border, and evaluate its statistical significance by comparison to 1000 random permutations.

To answer Research Question 2 – *Are population, cost of living, abortion laws, or support for education associated with being childfree* – we used two related approaches. First, we computed the simple bivariate association between the estimated percent a state’s population that is childfree and each of these state-level characteristics. Second, we estimated a generalized linear mixed-effects model with random intercepts using the lme4 package for R [36], which predicts the likelihood that an individual is childfree as a function of both individual and state characteristics, adjusting for the fact that individuals are nested in states and therefore are not independent observations. In this model, age, education, urbanicity, population, regional price parities, and per pupil spending were mean centered; political ideology and abortion rights scales were centered on their middle categories (‘moderate’ and ‘some restrictions/protections’, respectively). This model uses raw, unweighted data because demographic characteristics are already included as covariates [37].

Finally, to answer Research Question 3 – *What is the relative impact of individual-level and state-level characteristics on the likelihood that an individual is childfree* – we computed X-standardized coefficients in the mixed-effects model, then compared the effect sizes of individual demographic characteristics and state contextual characteristics.

The data used in this work come from the Civic Health and Institutions Project (chip50.org). The funders and the principal investigators of the CHIP50 Project bear no responsibility for the analyses reported here or the content of the work. The data and code necessary to reproduce the analyses reported in this manuscript are available in the Open Science Framework at https://osf.io/qexm3.

## Results

Before turning to the proposed analyses, we first validate these data by comparing prevalence estimates computed from them to previously published national and single-state prevalence estimates. First, a recent study using data from the National Survey of Family Growth found that 29.4% of non-parents age 18–44 in the U.S. were childfree in 2023 [3]. Using the CHIP50 data to examine the same subpopulation (i.e., non-parents age 18–44), we find that 31.2% are childfree, which is not statistically significantly different from the earlier estimate (z=−1.808, *p* = 0.071). Second, a recent study using data from the Michigan State of the State Survey found that 20.94% of adults in Michigan were childfree [15]. Using the CHIP50 data to examine the same subpopulation (i.e., Michigan residents of all ages), we find that 19.9% are childfree, which is not statistically significantly different from the earlier estimate (*z* = 0.477, *p* = 0.634). Because prevalence estimates derived from the CHIP50 data match previously published prevalence estimates derived from other sources at both the national and state levels, we proceed with our planned analyses.

Table 1 reports the estimated percent of adults who are childfree in each state. These values highlight substantial variation in the prevalence of childfree adults throughout the country, with childfree adults being twice as common in the highest prevalence states (California at 22.0% and Michigan at 19.9%) than in the lowest prevalence states (South Dakota at 10.0% and Louisiana at 9.4%).

**Table 1:** Estimated percent of adults who are childfree, by state.

State	N	%	SE	State	N	%	SE
California	944	22.00%	1.6	Oregon	362	16.40%	2.1
Michigan	472	19.90%	2.2	Missouri	384	16.40%	2.2
New Hampshire	357	19.40%	2.3	Oklahoma	375	16.30%	2.3
North Carolina	473	18.90%	2.1	New York	705	16.30%	1.7
Illinois	535	18.80%	2.0	Ohio	463	16.20%	2.0
Colorado	401	18.70%	2.2	West Virginia	329	15.80%	2.8
Massachusetts	374	18.40%	2.4	Alabama	375	15.80%	2.2
Pennsylvania	529	18.30%	1.9	Nebraska	346	15.50%	2.4
Washington	356	17.90%	2.2	Iowa	352	15.10%	2.4
New Jersey	432	17.90%	2.1	Mississippi	346	15.10%	2.6
Connecticut	305	17.80%	2.6	Hawaii	320	15.10%	2.2
Arizona	419	17.60%	2.3	Wyoming	253	14.90%	2.5
Rhode Island	342	17.40%	2.5	Vermont	219	14.20%	2.7
Maryland	413	17.30%	2.1	Kentucky	357	13.90%	2.2
Texas	770	17.20%	1.5	Montana	295	13.60%	2.3
Georgia	484	17.20%	2.0	Maine	348	13.20%	2.1
Virginia	356	17.00%	2.3	Arkansas	351	13.10%	2.1
Tennessee	386	16.90%	2.2	Utah	354	12.80%	2.0
Alaska	239	16.90%	2.9	Delaware	316	12.70%	2.2
Nevada	406	16.80%	2.3	South Carolina	349	12.10%	2.0
Wisconsin	394	16.70%	2.2	Kansas	368	11.60%	2.1
New Mexico	369	16.70%	2.5	Idaho	346	11.40%	1.9
Indiana	371	16.70%	2.2	North Dakota	282	10.10%	2.1
Florida	757	16.60%	1.5	South Dakota	282	10.00%	2.1
Minnesota	400	16.50%	2.0	Louisiana	357	9.40%	1.7

**Fig 1:**
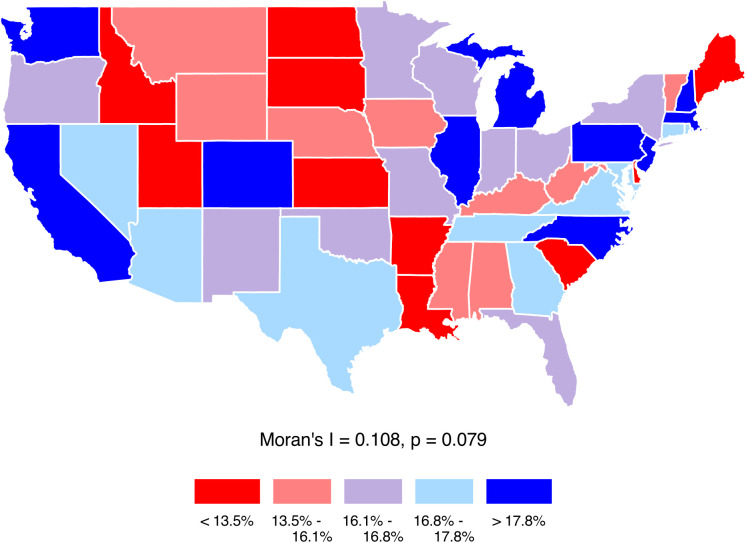
Percent of state population that is childfree, by quintile. Alaska (16.8%) and Hawaii (15.1%) are not shown, but are included in analyses.

Fig 1 maps these values, by quintile, in the contiguous 48 states and provides an answer to Research Question 1. Although several low-prevalence states were located in the Mountain West and Great Plains regions (e.g., Idaho, North Dakota, Kansas), high-prevalence states were more widely distributed (e.g., California, Illinois, Michigan, New York). A non-significant Moran’s I statistic indicates that there is no evidence that this population was clustered in specific multi-state regions (*I* = 0.108, *p* = 0.079; restricted to 48 contiguous states: *I* = 0.104, *p* = 0.097).

**Fig 2:**
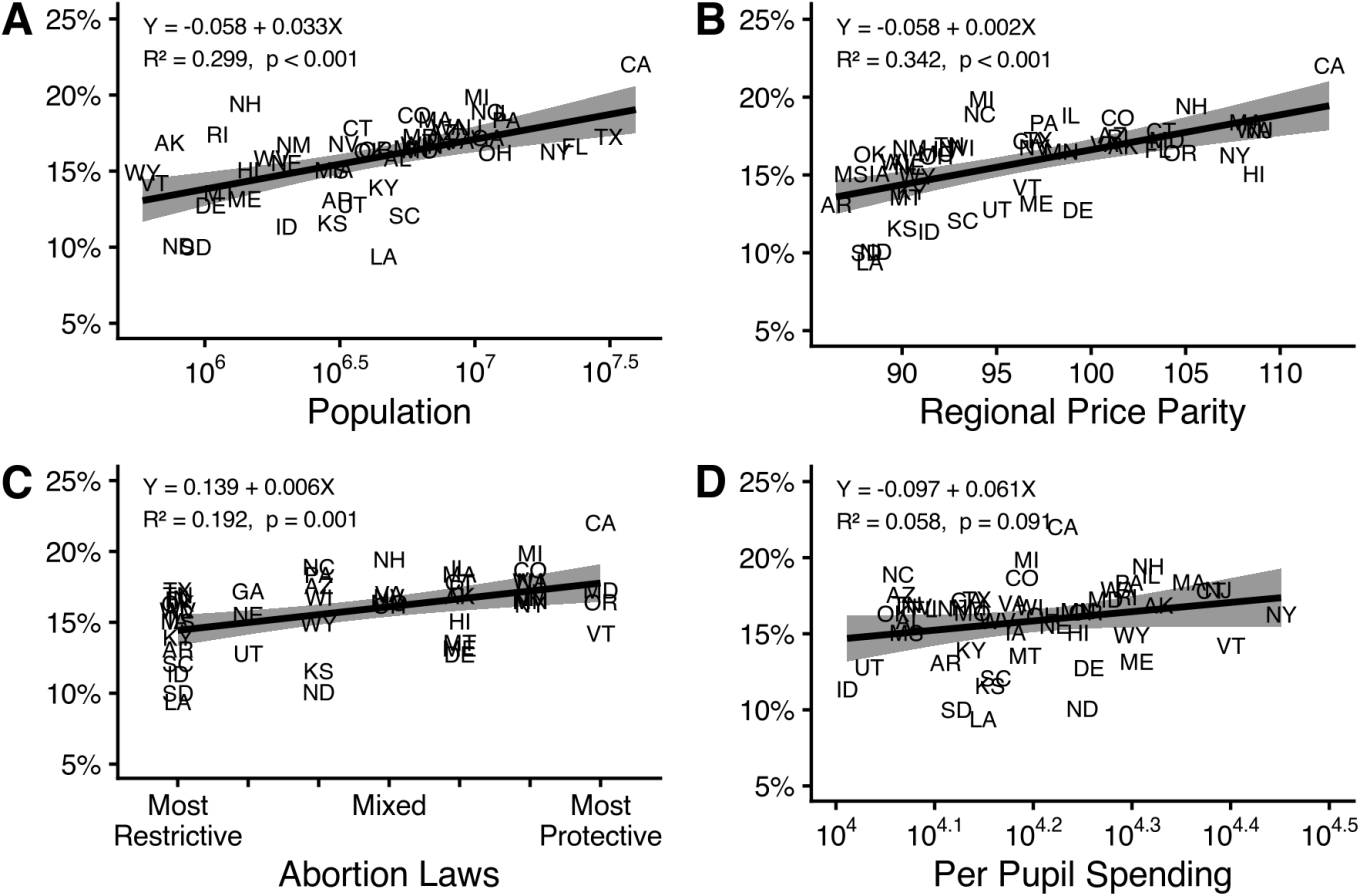
Association between state childfree prevalence and state (A) population, (B) regional price parity, (C) abortion laws, and (D) per pupil spending.

Fig 2 displays the bivariate association between the percent of each state’s population that was childfree and each of four state characteristics. Panel A indicates a statistically significant positive association between prevalence and population, such that a larger percent of adults were childfree in more populated states (*R*^2^ = 0.299, *p* < 0.001). Panel B indicates a statistically significant positive association between prevalence and cost of living, such that a larger percent of adults were childfree in states with a higher cost of living (*R*^2^ = 0.342, *p* < 0 001). Panel C indicates a statistically significant positive association between prevalence and abortion laws, such that a larger percent of adults were childfree in states with more legal protections for abortion (*R*^2^ = 0.192, *p* < 0.001). Finally, panel D indicates a non-significant association between education spending and prevalence (*R*^2^ = 0.058, *p* = 0.091). Collectively, these results provide a preliminary answer to Research Question 2: at the bivariate level, population, cost of living, and abortion laws were associated with the prevalence of childfree adults in a state.

Table 2 reports the estimates from a general linear mixed model predicting whether an individual was childfree as a function of individual and state characteristics. The intra-class correlation coefficient, which measures the fraction of total variance in prevalence that occurs at the state level, was nearly zero (*ICC* = 0.007). As a result, the variance of the state random effects was also nearly zero, and these results are nearly equal to those obtained from an ordinary least squares model.

**Table 2:** Estimates from a general linear mixed model predicting whether an individual was childfree as a function of individual and state characteristics (adults = 20,118; States = 50; ICC = 0.007).

	B	SE	p	β
Intercept	−2.658	0.053	< 0.001	−1.851
Individual characteristics				
Male	0.427	0.041	< 0.001	0.213
Single	1.259	0.045	< 0.001	0.579
Age	0.017	0.001	< 0.001	0.281
White	0.319	0.049	< 0.001	0.145
Liberal	0.131	0.013	< 0.001	0.209
Education	−0.001	0.02	0.948	−0.001
Urbanicity	0.021	0.015	0.168	−0.033
State characteristics				
Population	0.114	0.056	0.043	0.052
Regional Price Parities	0.011	0.005	0.023	0.076
Abortion Rights	0.025	0.014	0.081	0.054
Per Pupil Spending	−0.775	0.265	0.003	−0.082

β = X-standardized log odds.

At the individual level, a person was statistically significantly more likely to be childfree if they were male (*B* = 0.427, *p* < 0.001), single (*B* = 1.259, *p* < 0.001), older (*B* = 0.017, *p* < 0.001), White (*B* = 0.319, *p* < 0.001), and liberal (*B* = 0.131, *p* < 0.001). At the state level, a person was statistically significantly more likely to be childfree if they lived in a state with a larger population (*B* = 0.114, *p* = 0.043), a higher cost of living (*B* = 0.011, *p* = 0.023), and less support for public education (B=−0.775, *p* = 0.003). Because they estimate the independent effect of each characteristic controlling for the others, these results provide a more nuanced answer to Research Question 2: larger population and higher cost of living increase the likelihood of an individual being childfree, while greater support for public education decreases the likelihood of an individual being childfree.

Because they represent logged odds ratios (i.e., logits), the magnitudes of these effect sizes are difficult to interpret and cannot be compared to each other. The β column reports X-standardized log odds, which although still difficult to interpret, can be compared and thus provide an answer to Research Question 3. The standardized effects of individual characteristics on the likelihood of being childfree (range: 0.145–0.579) were much stronger than the standardized effects of state characteristics (range: −0.082–0.076). This suggests that although both individual and state characteristics may play a role in whether or not a given individual is childfree, the state context plays a much smaller role. This is consistent with past research focused on fertility [19,22,23].

**Fig 3:**
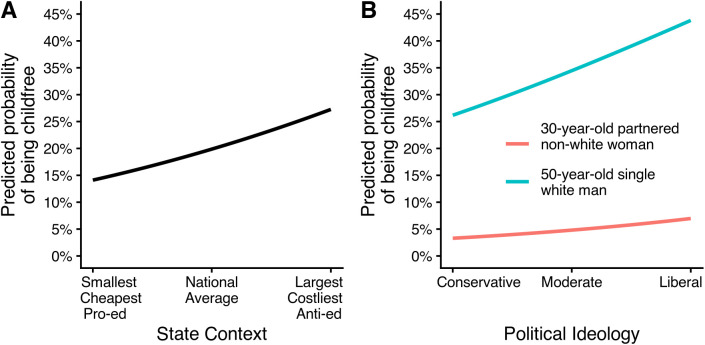
Predicted probability of (A) a politically moderate single white 30-year old female being childfree by state context, and (B) two demographically different adults being childfree, holding state population, cost of living, and education support constant at the national average.

To further facilitate interpretation, Fig 3 translates these estimates into predicted probabilities under a range of scenarios. Panel A illustrates the impact of state characteristics on the predicted probability of being childfree, holding individual characteristics constant. Specifically, it illustrates the predicted probability that a politically moderate single white 30-year old female is childfree, as a function of her state’s population, cost of living, and support for public education. For example, this model predicts that there is an 14.1% chance such a woman is childfree if she lives in the least populated, cheapest, most pro-education state, and a 27.2% chance she is childfree if she lives in the most populated, costliest, most anti-education state. That is, for an individual with these characteristics, variation in state context could shift the probability of being childfree by 13.1 percentage points.

Panel B illustrates the impact of individual characteristics on the predicted probability of being childfree, holding state characteristics constant at the national average. For example, this model predicts that there is 3.3% chance a 30-year-old conservative partnered non-white woman living in an average state will be childfree. In contrast, it predicts that there is a 43.8% chance that a 50-year-old liberal single white man living in an average state will be childfree. That is, for individuals living in an average state, these variations in individual characteristics should shift the probability of being childfree by 40.5 percentage points. Collectively, these illustrative scenarios demonstrate that although both individual and state characteristics are associated with the probability that a person is childfree, individual characteristics matter more than state context.

## Discussion

In this study, we used CHIP50 data to identify childfree adults (i.e., adults who do not have or want children) in samples weighted to be representative of each state in the U.S. We then used these data to examine the spatial distribution of the U.S. childfree population (Research Question 1), and to examine the individual and contextual characteristics that are associated with being childfree (Research Questions 2 and 3). It is among the first studies to bring a spatial and cross-regional comparative perspective to studying this group, and thus opens new avenues for research on this large and growing population.

Research Question 1 asked how the U.S. childfree population is spatially distributed, and whether this population is concentrated in certain areas. We observed substantial variation in state-level prevalence of childfree adults, ranging from 22% in California to 9.4% in Louisiana. However, we did not observe evidence of multi-state regional clustering in the spatial distribution of the childfree population. Many low-prevalence states are located in the Mountain West, but high-prevalence states are scattered throughout the country. These results suggest that being childfree is not a regional phenomenon, and that large populations of childfree adults exist throughout the country.

Research Question 2 asked whether state characteristics are associated with being childfree. Focusing on simple bivariate associations at the state level, childfree adults were more common in more populated states with higher costs of living and greater protections for abortion. However, controlling for individual and state characteristics, only larger state population and higher cost of living were associated with an increased likelihood of being childfree. In contrast, greater spending on public education was associated with a lower likelihood of being childfree. Across these two analyses, two state characteristics had a consistent positive association: population and cost of living. This suggests that individuals were more likely to be childfree when they lived in states where raising children would be expensive, and where there are already many other childfree people.

Research Question 3 asked whether individual-level demographic characteristics or state-level contextual characteristics are more closely associated with being childfree. We found that, although a state’s population and cost of living are associated with the likelihood of being childfree, these associations were relatively weak compared to individual demographic characteristics. For example, the association between being childfree and being single was 11 times stronger than the association between being childfree and a state’s population. This finding has important policy implications, or rather, policy non-implications. State policies designed to achieve population growth and encourage people to have children by, for example, reducing the cost of living or improving public education, are unlikely to have an effect on people who do not want children [38]. Importantly, although non-significant and weak associations can be ‘ruled out’ as possible policy levers, significant strong associations cannot necessarily be ‘ruled in’ as possible policy levers because their causal direction is unknown. Therefore, these results do not imply that state policies to, for example, encourage marriage could be used to deter childfreeness.

This study has a number of strengths, including reliance on a large sample that is weighted to be representative of each state, simultaneous consideration of individual and state-level characteristics, and shared data and materials for transparency. However, these results are also subject to some limitations, which highlight opportunities for future research. First, although it is among the first spatially-explicit studies of the childfree population, these data allow only the relatively coarse state level of spatial resolution. Future studies of the spatial characteristics of the childfree population should explore opportunities to collect finer-grained location data, which may reveal spatial clustering at other scales. Second, as cross-sectional data, these analyses only permit the identification of associations, but do not allow the estimation of causal effects. Future studies should explore collecting repeated cross-sectional data to identify regional trends [3], and panel data to identify causal effects and within-person changes over the life course [39]. Third, as one of the first studies of potential state-level contextual effects, this exploratory study examined a small subset of potential contextual factors (population, abortion policy, education spending, cost of living). Future studies may consider other contextual effects (e.g., political party of the state’s governor), and may also explore possible compositional effects (e.g., fraction of the state’s population that is Republican), which are likely closely associated with some factors already included in these models (e.g., abortion policy). Finally, although these analyses use weights to adjust quota-sampled data to be representative with respect to race, ethnicity, age, gender, education, and geographic region, these data may still not be representative with respect to other characteristics.

The childfree population in the U.S. is both large and growing, but relatively little is known about where childfree people live. This study used data from all 50 states to examine this population’s spatial distribution in the U.S., and to examine state-level characteristics associated with being childfree. Childfree adults are common in some states like California, and rare in others like Louisiana, but there is little evidence that this population is clustered in specific multi-state regions. Additionally, although a state’s population and cost of living are positively associated with being childfree, these associations are relatively weak. Whether an individual is childfree is more closely associated with their demographic characteristics (e.g., being single, older, male) than with characteristics of their state, which limits the potential effectiveness of state policy designed to encourage population growth through larger families. These findings set the stage for future research on the spatial features of and contextual influences on this emerging population.
