# Supplementary material for: The spatial distribution of the U.S. childfree population: Does state context matter?
Source: PLoS One. 2026 Aug 12;21(8):e0352872. doi: 10.1371/journal.pone.0352872 (PMC13465808; doi:10.1371/journal.pone.0352872)
Supplement: Supplementary file 1 [file pone.0352872.s002.pdf]

Summary of Revisions to "The Spatial Distribution of the U.S. Childfree Population: Does State Context Matter?" (PONE-D-25-63252)

Dear Dr. Yu,

Thank you for obtaining these very helpful reviewer comments, and for the opportunity to submit a revised version of this manuscript. In this cover letter we briefly summarize the revisions we made to address the reviewers' feedback.

1 Editor

1.1 "Please ensure that your manuscript meets PLOS ONE's style requirements, including those for file naming."

We have reviewed the formatting requirements for PLOS One, and have ensured that the manuscript, figures, and file names are in compliance.

1.2 "Please note that your Data Availability Statement is currently missing the repository name. If your manuscript is accepted for publication, you will be asked to provide these details on a very short timeline. We therefore suggest that you provide this information now, though we will not hold up the peer review process if you are unable."

We have updated our Data Availability Statement to include the name of the repository: Open Science Framework. The complete Data Availability Statement now reads: "*The data and code necessary to reproduce the analyses reported in this manuscript are available in the Open Science Framework at https://osf.io/qexm3.*"

1.3 "We note that Figure 1 in your submission contain map/satellite images which may be copyrighted. We require you to either (1) present written permission from the copyright holder to publish these figures specifically under the CC BY 4.0 license, or (2) remove the figures from your submission."

The coordinates used to draw state borders was obtained from the public domain Natural Earth project. The terms of use located at the above link specify: "No permission is needed to use Natural Earth. Crediting the authors is unnecessary."

2 Reviewer #1

2.1 "The manuscript addresses an important and understudied topic in demographic research. The geographic distribution of voluntary childlessness has received limited attention compared with other dimensions of fertility behavior. The dataset used appears substantial and potentially valuable."

We are happy to hear that you found the topic important, and agree that these data are helpful for advancing this line of research.

2.2 "The manuscript defines childfree individuals as those who 'do not have and do not want children.' However, the operationalization of this concept is not sufficiently explained. The manuscript does not clarify the exact survey items used to determine childfree status, whether respondents could express uncertainty, and how individuals who might want children later were classified. This is a critical issue because the literature distinguishes between childfree (voluntarily childless), childless (involuntary or circumstantial), and not-yet-parents Without a transparent classification procedure, the results may be difficult to interpret. My opinion is that the authors should provide the exact survey question wording, the response options, and the decision rule used for classification."

Our operationalization of childfree individuals specifically captures individuals who do not have and do not want children. Not-yet-parents, undecided, and childless individuals were all classified as not childfree.

In the 'variables: childfree status' subsection, we have expanded our discussion of the operationalization of childfree status: "*The CHIP50 survey followed an existing measurement framework (Neal and Neal, 2024) for measuring respondents' family status. First, all respondents were asked "Do you have, or have you ever had, any biological or adopted children?" and could respond yes, no, don't know, or prefer not to answer. Second, respondents who answered 'no' to the first question were asked "Do you plan to have any biological or adopted children in the future?" and again could respond yes, no, don't know, or prefer not to answer. Finally, respondents who answered 'no' to the second question were asked "Do you wish you had, or could have, biological or adopted children?" and again could respond yes, no, don't know, or prefer not to answer. Respondents who answered 'no' to all three questions were classified as childfree, while respondents who answered 'yes' or 'don't know' to any question were classified as not childfree. In this study, respondents' childfree status was measured using a binary indicator variable equal to 1 for childfree respondents, and 0 for respondents who are not childfree.*"

2.3 "The manuscript repeatedly describes the dataset as 'representative,' but no evidence is provided to support this claim. Specifically, the methods section does not report the sampling frame, the recruitment strategy, the survey mode (online panel, telephone survey, etc.), the response rate, and the weighting procedures. Given that the dataset includes respondents from all 50 states, differences in state sample size could strongly affect prevalence estimates. At minimum, the authors should report: sample size by state, weighting strategy (if used), and demographic comparisons to census benchmarks. Without this information, it is difficult to evaluate the reliability of the geographic estimates."

Thank you for this helpful comment. We have addressed it in the revised manuscript with four changes. First, in the methods section, we now explain that "*To obtain state-level representative samples, they [the CHIP50 survey team] collected data using quota sampling in each state, then computed state-specific post-stratification weights with respect to race, ethnicity, age, gender, education, and geographic region.*" Second, in Table 1 we report both the sample size for each state, and the standard error of each state's prevalence estimate. Third, we have replaced claims of the data's representativeness with the more accurate and descriptive explanation that the data are "*weighted to be representative of each state*." Finally, we have added a new data validation

step in which we validate these data by comparing both national and state-specific prevalence estimates to previously published estimates. We describe this new data validation step in the first paragraph of the 'Analysis Plan' section, and in the first paragraph of the 'Results' section.

2.4 "States are extremely large geographic units and may obscure meaningful spatial variation occurring at smaller scales (e.g., metropolitan areas). For example, it is plausible that childfree populations are concentrated in large metropolitan regions, even if state-level differences appear modest. The manuscript should acknowledge that the state level may be too coarse to detect clustering."

We note this as a limitation of the study: "*although it is among the first spatially-explicit studies of the childfree population, these data allow only the relatively coarse state level of spatial resolution. Future studies of the spatial characteristics of the childfree population should explore opportunities to collect finer-grained location data.*"

2.5 "Although the manuscript refers to spatial distribution, it does not conduct any formal spatial clustering or spatial autocorrelation analysis. Terms such as "clustering" should therefore be used cautiously unless supported by methods such as: Moran's I, Spatial Regression, and Geographically Weighted Regression."

We do perform a formal test of clustering using Moran's I. In the analysis plan, we wrote: "*To answer RQ1 we first computed the percent of each state's population that was childfree using state-specific post-stratification survey weights. We then computed Moran's I, where spatial proximity is defined by a shared border, and evaluate its statistical significance by comparison to 1000 random permutations.*" In the results section, we wrote: "*A non-significant Moran's I statistic indicates that there is no evidence that this population was clustered in certain areas ($I = 0.108$, $p = 0.079$; restricted to 48 contiguous states: $I = 0.104$, $p = 0.097$).*" Finally, in Figure 1, we wrote: "*Moran's I = 0.108, p = 0.079*".

2.6 "The study includes contextual variables such as state population size and cost of living, but the theoretical justification for these choices is limited. Other potentially relevant contextual factors are not considered, including: Political ideology of the state, religious composition, urbanization level, family policy environment, housing affordability, and educational attainment levels. The manuscript should clarify whether these variables were considered and explain the rationale for the final selection."

We address these issues in the subsection titled "The role of state context",:

- **Rationale for variable selection:** "*The choice of characteristics was informed by past fertility and childfree research cited below. It was also informed by a desire to focus on genuinely contextual features, as opposed to variables that are merely aggregations of individual-level characteristics (e.g., percent White) that can be more precisely modeled at the individual level (e.g., race).*"

- **Justification for population size:** "*...through a common subcultural formation mechanism (Fischer, 1975), total state population may be associated with being childfree. Larger states (and denser localities) have, on average, more visible childfree populations. These*

more visible populations not only offer more community and support to existing childfree people, but may also lead others to identify as childfree by normalizing the choice not to have children."

- **Justification for cost of living:** "*Among the most salient economic conditions is the cost of living, which may be associated with being childfree. Research in the U.S. (Minkin et al., 2024) and globally (United Nations Fund for Population Activities, 2025) has consistently found that the costs of raising children, and other economic concerns, are among the most commonly cited reasons for not having children. For example, in 2024, 36% of U.S. non-parents reported that their inability to "afford to raise a child" was among the major reasons they were unlikely to have children in the future.*"

- **Justification for abortion laws:** "*state abortion laws may be associated with being childfree because they shape the local risks involved in being pregnant or needing to obtain reproductive health care. Following the U.S. Supreme Court's ruling in Dobbs v. Jackson, which overturned Roe v. Wade and allowed states to enforce their own restrictions on abortion, the number of people identifying as childfree (Neal and Neal, 2024) and pursuing voluntary sterilization (Xu et al., 2024) increased in states with fewer protections.*"

- **Justification for education spending:** "*state support for public education may be associated with being childfree. There is limited prior research to support this possibility. However, we consider it because spending on public education is among the more visible ways that the state can invest in children, and signal support for parents and parenting. It is also more directly controlled by state policy than the other three characteristics, and thus if associated with being childfree, would have more direct policy implications.*"

- **Other possible variables:** We appreciate your suggestion of other possible variables. We believe that we already consider 'family policy environment' by including abortion policy, and we believe we already consider 'housing affordability' by including cost of living. We do not consider political ideology, urbanization, or education at the state level because these are individual characteristics that we already model at the individual level. Finally, unfortunately, individuals' religious affiliation is not available in these data.

2.7 "The statistical analysis relies on mixed-effects regression models, which is an appropriate methodological approach. However, the description of the models is insufficient. The manuscript should clearly report: Whether the models include random intercepts or random slopes, the estimated variance components, the intraclass correlation coefficient (ICC), diagnostics for model assumptions, and model comparison statistics. Providing this information would greatly improve the transparency and reproducibility of the analysis."

We have addressed this comment in the revised manuscript in three ways. First, in the analysis plan, we now explain that the model is fitted "*with random intercepts*". Second, in the results section, we now explain that "*The intra-class correlation coefficient, which measures the presence of clustering by state, was nearly zero ($ICC = 0.007$). As a result, the variance of the state random effects was also nearly zero, and these results are nearly equal to those obtained from an ordinary least squares model.*" Finally, we share all of the raw data and replication code so that interested users can reproduce these findings as well as consider alternative specifications.

2.8 "The manuscript does not explicitly state whether the research received ethical approval from an Institutional Review Board (IRB). Because the study involves survey data from human respondents, the manuscript must include: IRB approval or exemption, confirmation of informed consent, and description of data privacy protections. This information is required for compliance with the ethical standards of PLOS ONE."

We provide details about IRB review in the third paragraph of the methods section: "*The Michigan State University Institutional Review Board determined that analysis of these data does not constitute human subjects research because the data are anonymous and publicly available (STUDY00009134).*"

We have also provided the editor with the PLOS Human Subjects Research checklist and documentation from the IRB.

2.9 "The manuscript does not include a Data Availability Statement. Since PLOS ONE requires open or accessible data where possible, the authors should specify: Whether the dataset is publicly available, where it can be accessed, whether the analysis code will be shared. If the data cannot be made public, the authors must explain the restrictions."

We have provided the following Data Availability Statement: "*The data used in this work come from the Civic Health and Institutions Project (chip50.org). The funders and the principal investigators of the CHIP50 Project bear no responsibility for the analyses reported here or the content of the work. The data and code necessary to reproduce the analyses reported in this manuscript are available in the Open Science Framework at https://osf.io/qexm3.*"

2.10 "ABSTRACT: 'Childfree adults represent a large and growing fraction of the total population.' This claim requires a citation. It is unclear whether the growth of the childfree population has been empirically established at the national level."

PLOS One does not allow citations in the abstract. We also make this claim in the first three sentences of the manuscript, where it is accompanied by three supporting citations: National Survey of Family Growth (2019), National Survey of Family Growth (2022), and Neal and Neal (2025).

2.11 "ABSTRACT: 'Prevalence ranges from 22% in California to 9.4% in Louisiana.' Please clarify whether these estimates are weighted, and whether confidence intervals were calculated. Without uncertainty estimates, comparisons between states may be misleading."

The abstract is intended to provide a brief overview of the study, with major findings. Providing the requested information in the abstract would exceed the maximum word count permitted for the abstract. However, these details are provided in the body of the manuscript.

2.12 "INTRODUCTION: 'Relatively little is known about where childfree adults live.' This statement somewhat overstates the gap in the literature. Prior research has examined regional variation in fertility preferences and childlessness. The authors should more precisely explain how their contribution differs from previous studies."

Although there is research on regional variation in fertility preferences (e.g., how many children people have or want to have) or childlessness (i.e., people who wanted children but could not have them), we are not aware of any prior spatially explicit research on childfree individuals.

2.13 "The manuscript should report the distribution of respondents across states. Some states may have very small sample sizes, which would reduce the reliability of prevalence estimates."

Table 1 now reports the sample size for each state. Table 1 also reports the Standard Error for each state's prevalence estimate, which provides an indicator of the estimate's error.

We have also added a new data validation step in which we validate these data by comparing both national and state-specific prevalence estimates to previously published estimates. We describe this new data validation step in the first paragraph of the 'Analysis Plan' section, and in the first paragraph of the 'Results' section.

2.14 "Use of the term 'representative'. Unless the sampling design supports national representativeness, the authors should replace this term with 'large multi-state survey sample.'

We have clarified the survey's sampling design and clarified our use of the term 'representative' (see 2.3).

2.15 "The manuscript should include the exact survey questions used to determine the parental status, and the fertility intentions."

We now provide this information in the 'variables: childfree status' subsection (see 2.2).

2.16 "RESULTS: 'The childfree population is not clustered geographically.' This conclusion is not fully supported by the analysis because firstly, only state-level variation is examined and secondly no formal spatial clustering tests are conducted. The statement should therefore be moderated."

In all cases where we discuss clustering, we now clarify that we are focused on clustering "*within multi-state regions*". We conduct a formal test of clustering, which we report in the results section: "*A non-significant Moran's I statistic indicates that there is no evidence that this population was clustered in multi-state regions (*$I = 0.108$, $p = 0.079$*; restricted to 48 contiguous states:* $I = 0.104$, $p = 0.097$*).*"

2.17 "The state prevalence table should include sample size per state, standard errors, confidence intervals, and indication of whether estimates are weighted."

Table 1 reports the sample size for each state, and the standard errors for the prevalence estimates. Due to space limitations, and because they can be computed directly from the information provided by readers at their preferred confidence level, we do not to report confidence intervals in this table. In this analysis plan, we explain that "*we...computed the percent of each state's population that was childfree using state-specific post-stratification survey weights.*"

2.18 "The state geographic distribution figure would benefit from a clearer legend, an explanation of color intervals, and an indication of uncertainty."

We are unsure how to improve the legend of Figure 1. The legend and caption already explain that the color intervals represent quintiles, and the uncertainty of each prevalence estimate is already reported in Table 1 in the form of the estimate's standard error.

2.19 "The manuscript briefly mentions policy implications but does not elaborate on them. It would be helpful to clarify why understanding the spatial distribution of the childfree population is relevant for policy."

We do not believe that understanding the spatial distribution (Research Question #1) has direct policy implications. Instead, as we explain at the end of the background section, we believe the answer to Research Question #3 – Are individual-level demographic characteristics or state-level contextual characteristics more closely associated with being childfree – has policy implications. Specifically, we write: "*Answering this question is important for understanding the potential effectiveness of state policy intended to encourage residents to have children by, for example, lowering the cost of living or boosting funding for education.*"

In the discussion section, when discussing Research Question #3, we return to these policy implications: "*This finding has important policy implications, or rather, policy non-implications. State policies designed to achieve population growth and encourage people to have children by, for example, reducing the cost of living or improving public education, are unlikely to have an effect on people who do not want children (Gietel-Basten et al., 2022). Importantly, although non-significant and weak associations can be 'ruled out' as possible policy levers, significant strong associations cannot necessarily be 'ruled in' as possible policy levers because their causal direction is unknown. Therefore, these results do not imply that state policies to, for example, encourage marriage could be used to deter childfreeness.*"

2.20 "The limitations section should explicitly mention the potential misclassification of fertility intentions, the cross-sectional design, and the coarse geographic aggregation."

We have expanded our discussion of the operationalization of childfree status to explain how it distinguishes childfree individuals from other types of non-parents (see 2.2).

We explicitly mention the other two limitations, together with future directions: "*First, although it is among the first spatially-explicit studies of the childfree population, these data allow only the relatively coarse state level of spatial resolution. Future studies of the spatial characteristics*

of the childfree population should explore opportunities to collect finer-grained location data. Second, as cross-sectional data, these analyses only permit the identification of associations, but do not allow the estimation of causal effects. Future studies should explore collecting repeated cross-sectional data to identify regional trends, and panel data to identify causal effects and within-person changes over the life course."

2.21 "Several sentences in the introduction are overly long and could be simplified."

We have aimed to simplify the writing in this revision.

2.22 "Some references appear to be missing publication years."

We have ensured that all references also include the publication year.

2.23 "Acronyms should be defined at first use."

We have ensured that all acronyms are defined when they are first used.

3 Reviewer #2

3.1 "There were a number of strengths of this research. First, this research provides a valuable insight into the spatial distribution of childfree people across the United States. Understanding where childfree people live is useful not only for researchers who seek to further explore the nature and characteristics of the childfree, but also for policy makers who would benefit from a greater understanding of fertility intentions, rather than just fertility behavior. This research also expands the literature surrounding the individual characteristics of childfree people; a growing yet understudied population."

We are very pleased to hear that you found many strengths in the work.

3.2 "Total state population, rather than state-level population density, is included as a state-level variable. The researchers explain that they do not believe population density to be relevant at the state level, but that total state population may be important for the likelihood of being childfree through a subcultural formation mechanism. However it is not clear how this mechanism may facilitate being childfree. The researchers should therefore briefly expand on this section of the introduction by explaining the subcultural formation process, as well as a short discussion of any relevant literature on childfree visibility by state population size, or why they believe the visibility of the childfree population may vary as a function of total state population specifically, rather than more local population density (i.e. why a larger, but less dense state may have a more visible childfree population than a smaller but more dense state)."

Thanks for highlighting that this was unclear. We are not aware of any prior research on childfree visibility by state population. But, we have re-written our rationale for considering state population to clarify our thinking: "*First, fertility research has found that non-parenthood is associated with local population density (Nisén et al., 2025). Measuring population density at the state level*

can be misleading because many states have significant rural and urban variation (e.g., New York City vs. the rest of New York state). However, total state population may also be associated with being childfree. For a given prevalence rate, states with larger populations will contain larger numbers of childfree individuals. These larger numbers of childfree individuals in the same state may not only offer community and support to existing childfree people, but may also lead others to identify as childfree by normalizing the choice not to have children." It is not possible to determine *why* state population is associated with being childfree, but we do find statistical support for this association both at the bivariate level with state prevalence (see Figure 2A) and at the multivariate level with individual choices (see Table 2).

3.3 "Related to the previous comment, urbanicity, which approximately captures the population density of the area where an individual lives, was included as an individual-level variable. Individual-level urbanicity was not a significant predictor of being childfree, but state-level population size was. These results challenge the expectation that childfree people are concentrated in more urban areas. This assumption is mentioned in the introduction, however, a discussion of the results surrounding total population and urbanicity should be included to explain why the researchers believe that total state population, rather than the population density of the specific area where a person lives, would be predictive of being childfree."

We do not have the expectation that childfree people are concentrated in urban areas, and do not make this assumption. Instead, we observe that "*findings about the relationship between urbanicity and being childfree have been mixed. Although one study found that childfree individuals were more likely to live in an urban area compared to parents (Simon et al., 2025), a review of nine studies found that childfree individuals were not more likely to live in an urban area (Jin et al., 2023).*" Because the findings have previously been mixed, we included urbanicity in our model with the hope of clarifying whether such an association exists. Our findings are consistent with past research suggesting there is no association. Because this variable's association is not significant, we refrain from discussing it further.

We do find that total state population is statistically significantly associated with being childfree. However, the key finding in Research Question #3 is that, despite being statistically significant, it has a relatively small effect compared to individual demographic characteristics such as sex or marital status. Because total state population has such a small effect, we do not believe there is much further to discuss about it.

3.4 "This study uses the CHIP50 dataset, which is a large, multi-university, representative sample that spans across all 50 states. Readers unfamiliar with CHIP50 may benefit from a short description of the broader purpose of this project or the types of variables included, and how participants were recruited to take part."

CHIP50 is an on-going survey project (with which we are not directly involved), and not a single dataset. In this study, we use public data from the 33rd wave of the CHIP50 project.

For readers unfamiliar with the CHIP50 project, we have expanded our description: "*The CHIP50 is a multi-university collaborative effort to collect data from non-probability quota samples that are weighted to be representative of the U.S. as a whole, and of each state, thereby permitting*

inferences at multiple geographic levels. It is the successor to the earlier 'COVID States Project,' which began collecting data in April 2020 to examine COVID-related health and policy. Each wave of the survey now includes core demographic questions accompanied by a unique set of researcher-generated questions in multiple fields including political science (Baum et al., 2024), public health (Santillana et al., 2024) and mental health (Perlis et al., 2021)." We have also expanded our description of their methodology: "*To obtain state-level representative samples, they recruited quota samples from online respondent panels, collected data using the PureSpectrum online polling platform, then computed state-specific post-stratification weights with respect to race, ethnicity, age, gender, education, and geographic region.*"

3.5 "Individual-level variables such as age, gender, race, relationship status, and political orientation were all found to be significant predictors of being childfree. These effects were much larger than the effects of state-level variables. Because of the importance of these individual-level variables for predicting the likelihood that a person will be childfree, a discussion of the role of individual differences in fertility intentions, specifically the choice to be childfree, should be included in the introduction when introducing Research Question #3. Specifically, the researchers explain why they chose each of the four state-level variables that were included in analysis, and so should also provide justification for including age, gender, race, relationship status, political orientation, education, and urbanicity specifically as individual-level variables."

We selected these variables because past studies have investigated their association with being childfree. They are intended only as control variables, not variables of direct theoretical interest. We included them in our model only to evaluate whether state-level characteristics have additional explanatory power beyond demographic control variables that are typically used in this literature. When introducing Research Question #3, we now write: "*Past research has investigated whether being childfree is associated with an individual's sex, marital status, age, race, political ideology, education, and the urbanicity of their residence (Simon et al., 2025; Jin et al., 2023; Neal and Neal , 2021, 2023; Neal and Neal, 2024).*"

3.6 "In panel 1 of Figure 3, only population size, cost of living, and educational spending are included in the predictive model. Abortion protections were found to significantly predict being childfree, so it is unclear why it was not included in the predictive probability analysis."

We find that Abortion Rights are significantly associated, at the bivariate level, with state prevalence of childfree adults. However, in the general linear model reported in Table 2, we *do not* find that Abortion Rights are statistically significantly associated with whether a given individual is childfree ($p = 0.081$). Therefore, we exclude this variable from the comparison of the predicted probability of an individual being childfree under varying state contexts shown in Figure 3A.

References

Baum, M. A., Druckman, J. N., Simonson, M. D., Lin, J., and Perlis, R. H. (2024). The political consequences of depression: How conspiracy beliefs, participatory inclinations, and depression affect support for political violence. *American Journal of Political Science*, 68(2):575–594.

Fischer, C. S. (1975). Toward a subcultural theory of urbanism. *American Journal of Sociology*, 80(6):1319–1341.

Gietel-Basten, S., Rotkirch, A., and Sobotka, T. (2022). Changing the perspective on low birth rates: Why simplistic solutions won't work. *The British Medical Journal*, 379.

Jin, C., Xu, X., Tooth, L. R., and Mishra, G. D. (2023). Voluntary childlessness: demographic, socio economic, family, and psychosocial factors. *Handbook of Labor, Human Resources and Population Economics*, pages 1–26.

Minkin, R., Horowitz, J., and Aragão, C. (2024). The experiences of U.S. adults who don't have children.

National Survey of Family Growth (2019). Listing C - Key Statistics accessed at https://www.cdc.gov/nchs/nsfg/key_statistics/c.htm.

National Survey of Family Growth (2022). Listing C - Key Statistics accessed at https://www.cdc.gov/nchs/nsfg/key_statistics/c-keystat.htm.

Neal, J. W. and Neal, Z. P. (2024). Prevalence of childfree adults before and after Dobbs v. Jackson in Michigan (USA). *PLOS One*, 19(1):e0294459.

Neal, J. W. and Neal , Z. P. (2021). Prevalence and characteristics of childfree adults in Michigan (USA). *PLOS One*, 16(6):e0252528.

Neal, J. W. and Neal , Z. P. (2023). Prevalence, age of decision, and interpersonal warmth judgements of childfree adults: Replication and extensions. *PLOS One*, 18(4):e0283301.

Neal, J. W. and Neal , Z. P. (2025). Tracking types of non-parents in the United States. *Journal of Marriage and Family*.

Neal , Z. P. and Neal, J. W. (2024). A framework for studying adults who neither have nor want children. *The Family Journal*, 32(1):121–130.

Nisén, J., Klüsener, S., Dahlberg, J., Dommermuth, L., Jasilioniene, A., Kreyenfeld, M., Lappegård, T., Li, P., Martikainen, P., Neels, K., et al. (2025). Economic conditions, education and parity-specific fertility: A sub-national regional study across 15 countries in Europe. *Population, Space and Place*, 31(6):e70074.

Perlis, R. H., Ognyanova, K., Quintana, A., Green, J., Santillana, M., Lin, J., Druckman, J., Lazer, D., Simonson, M. D., Baum, M. A., et al. (2021). Gender-specificity of resilience in major depressive disorder. *Depression and Anxiety*, 38(10):1026–1033.

Santillana, M., Uslu, A. A., Urmi, T., Quintana-Mathe, A., Druckman, J. N., Ognyanova, K., Baum, M., Perlis, R. H., and Lazer, D. (2024). Tracking COVID-19 infections using survey data on rapid at-home tests. *JAMA Network Open*, 7(9):e2435442–e2435442.

Simon, K. A., Meslay, G., Farr, R. H., and Russell, S. T. (2025). Demographic characteristics and parenthood across three cohorts of sexual minority adults. *Family Process*, 64(1):e70012.

United Nations Fund for Population Activities (2025). State of world population 2025.

Xu, X., Chen, L., Desai, V. B., Gross, C. P., Pollack, C. E., Schwartz, P. E., and Wright, J. D. (2024). Tubal sterilization rates by state abortion laws after the Dobbs decision. *Journal of the American Medical Association*, 332(14):1204–1206.
