# Supplementary material for: The spatial distribution of the U.S. childfree population: Does state context matter?
Source: PLoS One. 2026 Aug 12;21(8):e0352872. doi: 10.1371/journal.pone.0352872 (PMC13465808; doi:10.1371/journal.pone.0352872)
Supplement: Peer review history

# Peer review: Decision Letter 0

**DOI:** 10.1371/journal.pone.0352872.r001

**Authors:** Weijun Yu

15 Apr 2026

PONE-D-25-63252

The Spatial Distribution of the U.S. Childfree Population: Does State Context Matter?

PLOS One

Dear Dr. Neal,

Thank you for submitting your manuscript to PLOS ONE. After careful consideration, we feel that it has merit but does not fully meet PLOS ONE’s publication criteria as it currently stands. Therefore, we invite you to submit a revised version of the manuscript that addresses the points raised during the review process.

Please submit your revised manuscript by May 30 2026 11:59PM. If you will need more time than this to complete your revisions, please reply to this message or contact the journal office at plosone@plos.org. When you're ready to submit your revision, log on to https://www.editorialmanager.com/pone/ and select the 'Submissions Needing Revision' folder to locate your manuscript file.

Please include the following items when submitting your revised manuscript:

- A letter that responds to each point raised by the academic editor and reviewer(s). You should upload this letter as a separate file labeled 'Response to Reviewers'.
- A marked-up copy of your manuscript that highlights changes made to the original version. You should upload this as a separate file labeled 'Revised Manuscript with Track Changes'.
- An unmarked version of your revised paper without tracked changes. You should upload this as a separate file labeled 'Manuscript'.

If you would like to make changes to your financial disclosure, please include your updated statement in your cover letter. Guidelines for resubmitting your figure files are available below the reviewer comments at the end of this letter.

If applicable, we recommend that you deposit your laboratory protocols in protocols.io to enhance the reproducibility of your results. Protocols.io assigns your protocol its own identifier (DOI) so that it can be cited independently in the future. For instructions see: https://journals.plos.org/plosone/s/submission-guidelines#loc-laboratory-protocols. Additionally, PLOS ONE offers an option for publishing peer-reviewed Lab Protocol articles, which describe protocols hosted on protocols.io. Read more information on sharing protocols at https://plos.org/protocols?utm_medium=editorial-email&utm_source=authorletters&utm_campaign=protocols.

As the corresponding author, your ORCID iD is verified in the submission system and will appear in the published article. PLOS supports the use of ORCID, and we encourage all coauthors to register for an ORCID iD and use it as well. Please encourage your coauthors to verify their ORCID iD within the submission system before final acceptance, as unverified ORCID iDs will not appear in the published article. *Only*  the individual author can complete the verification step; PLOS staff *cannot*  verify ORCID iDs on behalf of authors.

We look forward to receiving your revised manuscript.

Kind regards,

Weijun Yu, Ph.D., M.D., M.S.

Academic Editor

PLOS One

Journal Requirements:

When submitting your revision, we need you to address these additional requirements.

1.Please ensure that your manuscript meets PLOS ONE's style requirements, including those for file naming. The PLOS ONE style templates can be found at

https://journals.plos.org/plosone/s/file?id=wjVg/PLOSOne_formatting_sample_main_body.pdf and

https://journals.plos.org/plosone/s/file?id=ba62/PLOSOne_formatting_sample_title_authors_affiliations.pdf

2. Please note that your Data Availability Statement is currently missing the repository name. If your manuscript is accepted for publication, you will be asked to provide these details on a very short timeline. We therefore suggest that you provide this information now, though we will not hold up the peer review process if you are unable.

3.We note that Figure 1 in your submission contain map/satellite images which may be copyrighted. All PLOS content is published under the Creative Commons Attribution License (CC BY 4.0), which means that the manuscript, images, and Supporting Information files will be freely available online, and any third party is permitted to access, download, copy, distribute, and use these materials in any way, even commercially, with proper attribution. For these reasons, we cannot publish previously copyrighted maps or satellite images created using proprietary data, such as Google software (Google Maps, Street View, and Earth). For more information, see our copyright guidelines: http://journals.plos.org/plosone/s/licenses-and-copyright.

We require you to either (1) present written permission from the copyright holder to publish these figures specifically under the CC BY 4.0 license, or (2) remove the figures from your submission:

1. You may seek permission from the original copyright holder of Figure 1 to publish the content specifically under the CC BY 4.0 license.

We recommend that you contact the original copyright holder with the Content Permission Form (http://journals.plos.org/plosone/s/file?id=7c09/content-permission-form.pdf) and the following text:

“I request permission for the open-access journal PLOS ONE to publish XXX under the Creative Commons Attribution License (CCAL) CC BY 4.0 (http://creativecommons.org/licenses/by/4.0/). Please be aware that this license allows unrestricted use and distribution, even commercially, by third parties. Please reply and provide explicit written permission to publish XXX under a CC BY license and complete the attached form.”

Please upload the completed Content Permission Form or other proof of granted permissions as an "Other" file with your submission.

In the figure caption of the copyrighted figure, please include the following text: “Reprinted from [ref] under a CC BY license, with permission from [name of publisher], original copyright [original copyright year].”

2. If you are unable to obtain permission from the original copyright holder to publish these figures under the CC BY 4.0 license or if the copyright holder’s requirements are incompatible with the CC BY 4.0 license, please either i) remove the figure or ii) supply a replacement figure that complies with the CC BY 4.0 license. Please check copyright information on all replacement figures and update the figure caption with source information. If applicable, please specify in the figure caption text when a figure is similar but not identical to the original image and is therefore for illustrative purposes only.

The following resources for replacing copyrighted map figures may be helpful:

USGS National Map Viewer (public domain): http://viewer.nationalmap.gov/viewer/

The Gateway to Astronaut Photography of Earth (public domain): http://eol.jsc.nasa.gov/sseop/clickmap/

Maps at the CIA (public domain): https://www.cia.gov/library/publications/the-world-factbook/index.html and https://www.cia.gov/library/publications/cia-maps-publications/index.html

NASA Earth Observatory (public domain): http://earthobservatory.nasa.gov/

Landsat: http://landsat.visibleearth.nasa.gov/

USGS EROS (Earth Resources Observatory and Science (EROS) Center) (public domain): http://eros.usgs.gov/#

Natural Earth (public domain): http://www.naturalearthdata.com/

4. If the reviewer comments include a recommendation to cite specific previously published works, please review and evaluate these publications to determine whether they are relevant and should be cited. There is no requirement to cite these works unless the editor has indicated otherwise.

Additional Editor Comments:

Please address all the comments raised by the two reviewers in your revised manuscript and rebuttal letter.

[Note: HTML markup is below. Please do not edit.]

Reviewers' comments:

Reviewer's Responses to Questions

**Comments to the Author**

1. Is the manuscript technically sound, and do the data support the conclusions?

The manuscript must describe a technically sound piece of scientific research with data that supports the conclusions. Experiments must have been conducted rigorously, with appropriate controls, replication, and sample sizes. The conclusions must be drawn appropriately based on the data presented.

Reviewer #1: Partly

Reviewer #2: Yes

**********

2. Has the statistical analysis been performed appropriately and rigorously?

Reviewer #1: No

Reviewer #2: Yes

**********

3. Have the authors made all data underlying the findings in their manuscript fully available?

The PLOS Data policy requires authors to make all data underlying the findings described in their manuscript fully available without restriction, with rare exception (please refer to the Data Availability Statement in the manuscript PDF file). The data should be provided as part of the manuscript or its supporting information, or deposited to a public repository. For example, in addition to summary statistics, the data points behind means, medians and variance measures should be available. If there are restrictions on publicly sharing data—e.g. participant privacy or use of data from a third party—those must be specified.

Reviewer #1: No

Reviewer #2: Yes

**********

4. Is the manuscript presented in an intelligible fashion and written in standard English?

PLOS ONE does not copyedit accepted manuscripts, so the language in submitted articles must be clear, correct, and unambiguous. Any typographical or grammatical errors should be corrected at revision, so please note any specific errors here.

Reviewer #1: Yes

Reviewer #2: Yes

**********

5. Review Comments to the Author

Please use the space provided to explain your answers to the questions above. You may also include additional comments for the author, including concerns about dual publication, research ethics, or publication ethics. (Please upload your review as an attachment if it exceeds 20,000 characters)

Reviewer #1: Overall Evaluation

Respected Authors,

This manuscript examines the geographic distribution of adults identifying as childfree in the United States and evaluates whether state-level contextual factors influence the likelihood of being childfree. Using a large survey dataset (N ≈ 20,118) and mixed-effects modeling, the authors conclude that individual demographic characteristics explain more variation than state-level context.

The manuscript addresses an important and understudied topic in demographic research. The geographic distribution of voluntary childlessness has received limited attention compared with other dimensions of fertility behavior. The dataset used appears substantial and potentially valuable.

However, several issues currently limit the manuscript’s contribution, such as the insufficient methodological transparency, the ambiguous operationalization of the key concept (“childfree”), the overinterpretation of state-level results, the limited discussion of survey representativeness, and the incomplete reporting of ethical approval and data availability

I think that substantial revisions are necessary before the manuscript meets the reporting and methodological standards required by PLOS ONE.

Major Comments

1. Conceptual Definition of “Childfree”

The manuscript defines childfree individuals as those who “do not have and do not want children.”

However, the operationalization of this concept is not sufficiently explained. The manuscript does not clarify the exact survey items used to determine childfree status, whether respondents could express uncertainty, and how individuals who might want children later were classified.

This is a critical issue because the literature distinguishes between childfree (voluntarily childless), childless (involuntary or circumstantial), and not-yet-parents

Without a transparent classification procedure, the results may be difficult to interpret. My opinion is that the authors should provide the exact survey question wording, the response options, and the decision rule used for classification.

2. Sampling Methodology and Representativeness

The manuscript repeatedly describes the dataset as “representative”, but no evidence is provided to support this claim. Specifically, the methods section does not report the sampling frame, the recruitment strategy, the survey mode (online panel, telephone survey, etc.), the response rate, and the weighting procedures. Given that the dataset includes respondents from all 50 states, differences in state sample size could strongly affect prevalence estimates.

At minimum, the authors should report: sample size by state, weighting strategy (if used), and demographic comparisons to census benchmarks. Without this information, it is difficult to evaluate the reliability of the geographic estimates.

3. Interpretation of Geographic Variation

The manuscript concludes that the childfree population is not strongly clustered geographically and that state-level context plays a limited role. This conclusion is problematic for two reasons.

3.1 State-Level Units Are Very Large

States are extremely large geographic units and may obscure meaningful spatial variation occurring at smaller scales (e.g., metropolitan areas). For example, it is plausible that childfree populations are concentrated in large metropolitan regions, even if state-level differences appear modest. The manuscript should acknowledge that the state level may be too coarse to detect clustering.

3.2 Lack of Spatial Analysis

Although the manuscript refers to spatial distribution, it does not conduct any formal spatial clustering or spatial autocorrelation analysis. Terms such as “clustering” should therefore be used cautiously unless supported by methods such as: Moran’s I, Spatial Regression, and Geographically Weighted Regression

4. Selection of State-Level Variables

The study includes contextual variables such as state population size and cost of living, but the theoretical justification for these choices is limited. Other potentially relevant contextual factors are not considered, including: Political ideology of the state, religious composition, urbanization level, family policy environment, housing affordability, and educational attainment levels. The manuscript should clarify whether these variables were considered and explain the rationale for the final selection.

5. Transparency of the Mixed-Effects Models

The statistical analysis relies on mixed-effects regression models, which is an appropriate methodological approach. However, the description of the models is insufficient. The manuscript should clearly report: Whether the models include random intercepts or random slopes, the estimated variance components, the intraclass correlation coefficient (ICC), diagnostics for model assumptions, and model comparison statistics. Providing this information would greatly improve the transparency and reproducibility of the analysis.

6. Ethical Approval and Research Integrity

The manuscript does not explicitly state whether the research received ethical approval from an Institutional Review Board (IRB). Because the study involves survey data from human respondents, the manuscript must include: IRB approval or exemption, confirmation of informed consent, and description of data privacy protections. This information is required for compliance with the ethical standards of PLOS ONE.

7. Data Availability

The manuscript does not include a Data Availability Statement. Since PLOS ONE requires open or accessible data where possible, the authors should specify: Whether the dataset is publicly available, where it can be accessed, whether the analysis code will be shared. If the data cannot be made public, the authors must explain the restrictions.

Specific Comments (Line-Level)

Abstract:

Sentence: “Childfree adults represent a large and growing fraction of the total population.” This claim requires a citation. It is unclear whether the growth of the childfree population has been empirically established at the national level.

Sentence: “Prevalence ranges from 22% in California to 9.4% in Louisiana.” Please clarify whether these estimates are weighted, and whether confidence intervals were calculated. Without uncertainty estimates, comparisons between states may be misleading.

Introduction

Statement: “Relatively little is known about where childfree adults live.” This statement somewhat overstates the gap in the literature. Prior research has examined regional variation in fertility preferences and childlessness. The authors should more precisely explain how their contribution differs from previous studies.

Methods:

Description of sample size (20,118 respondents). The manuscript should report the distribution of respondents across states. Some states may have very small sample sizes, which would reduce the reliability of prevalence estimates.

Use of the term “representative”. Unless the sampling design supports national representativeness, the authors should replace this term with “large multi-state survey sample.”

Classification of childfree respondents:

The manuscript should include the exact survey questions used to determine the parental status, and the fertility intentions.

Results:

Statement: “The childfree population is not clustered geographically.” This conclusion is not fully supported by the analysis because firstly, only state-level variation is examined and secondly no formal spatial clustering tests are conducted. The statement should therefore be moderated.

Tables:

State-level prevalence table: The table should include sample size per state, standard errors, confidence intervals, and indication of whether estimates are weighted.

Figures:

Map showing geographic distribution: The figure would benefit from a clearer legend, an explanation of color intervals, and an indication of uncertainty.

Discussion:

Policy implications: The manuscript briefly mentions policy implications but does not elaborate on them. It would be helpful to clarify why understanding the spatial distribution of the childfree population is relevant for policy.

Limitations: The limitations section should explicitly mention the potential misclassification of fertility intentions, the cross-sectional design, and the coarse geographic aggregation.

Minor Comments

1. Several sentences in the introduction are overly long and could be simplified.

2. Some references appear to be missing publication years.

3. Acronyms should be defined at first use.

Recommendation

Recommendation: Major Revision

The manuscript addresses an interesting and important research question and uses a large dataset that has the potential to make a meaningful contribution to demographic research on voluntary childlessness. However, substantial revisions are necessary, particularly regarding:

• methodological transparency,

• conceptual clarity,

• statistical reporting,

• ethical and data availability statements.

Addressing these issues would significantly strengthen the manuscript.

Reviewer #2: The purpose of this research was to explore the distribution of childfree people across the 50 states of the United States, and to examine state and individual-level characteristics that predict being childfree. The researchers used the CHIP50 dataset, a multi-university, representative sample of over 20,000 people across all 50 states to explore these research questions. Specifically, they utilize per-state percentages of childfree people, Moran’s I to assess region-level trends, and both bivariate associations and a generalized linear mixed-effects model to explore how the likelihood of being childfree can be predicted by state and individual characteristics. They also create predictive probabilities of being childfree to compare the effects of these state and individual characteristics.

There were a number of strengths of this research. First, this research provides a valuable insight into the spatial distribution of childfree people across the United States. Understanding where childfree people live is useful not only for researchers who seek to further explore the nature and characteristics of the childfree, but also for policy makers who would benefit from a greater understanding of fertility intentions, rather than just fertility behavior. This research also expands the literature surrounding the individual characteristics of childfree people; a growing yet understudied population.

Despite these strengths, there were several limitations that warrant discussion.

1. Total state population, rather than state-level population density, is included as a state-level variable. The researchers explain that they do not believe population density to be relevant at the state level, but that total state population may be important for the likelihood of being childfree through a subcultural formation mechanism. However it is not clear how this mechanism may facilitate being childfree. The researchers should therefore briefly expand on this section of the introduction by explaining the subcultural formation process, as well as a short discussion of any relevant literature on childfree visibility by state population size, or why they believe the visibility of the childfree population may vary as a function of total state population specifically, rather than more local population density (i.e. why a larger, but less dense state may have a more visible childfree population than a smaller but more dense state).

2. Related to the previous comment, urbanicity, which approximately captures the population density of the area where an individual lives, was included as an individual-level variable. Individual-level urbanicity was not a significant predictor of being childfree, but state-level population size was. These results challenge the expectation that childfree people are concentrated in more urban areas. This assumption is mentioned in the introduction, however, a discussion of the results surrounding total population and urbanicity should be included to explain why the researchers believe that total state population, rather than the population density of the specific area where a person lives, would be predictive of being childfree.

3. This study uses the CHIP50 dataset, which is a large, multi-university, representative sample that spans across all 50 states. Readers unfamiliar with CHIP50 may benefit from a short description of the broader purpose of this project or the types of variables included, and how participants were recruited to take part.

4. Individual-level variables such as age, gender, race, relationship status, and political orientation were all found to be significant predictors of being childfree. These effects were much larger than the effects of state-level variables. Because of the importance of these individual-level variables for predicting the likelihood that a person will be childfree, a discussion of the role of individual differences in fertility intentions, specifically the choice to be childfree, should be included in the introduction when introducing Research Question #3. Specifically, the researchers explain why they chose each of the four state-level variables that were included in analysis, and so should also provide justification for including age, gender, race, relationship status, political orientation, education, and urbanicity specifically as individual-level variables.

5. In panel 1 of Figure 3, only population size, cost of living, and educational spending are included in the predictive model. Abortion protections were found to significantly predict being childfree, so it is unclear why it was not included in the predictive probability analysis.

**********

6. PLOS authors have the option to publish the peer review history of their article (what does this mean?). If published, this will include your full peer review and any attached files.

If you choose “no”, your identity will remain anonymous but your review may still be made public.

**Do you want your identity to be public for this peer review?**  For information about this choice, including consent withdrawal, please see our Privacy Policy.

Reviewer #1: No

Reviewer #2: No

**********

[NOTE: If reviewer comments were submitted as an attachment file, they will be attached to this email and accessible via the submission site. Please log into your account, locate the manuscript record, and check for the action link "View Attachments". If this link does not appear, there are no attachment files.]

To ensure your figures meet our technical requirements, please review our figure guidelines: https://journals.plos.org/plosone/s/figures

You may also use PLOS’s free figure tool, NAAS, to help you prepare publication quality figures: https://journals.plos.org/plosone/s/figures#loc-tools-for-figure-preparation.

NAAS will assess whether your figures meet our technical requirements by comparing each figure against our figure specifications.

# Author comment: Author response to Decision Letter 1

**DOI:** 10.1371/journal.pone.0352872.r002

23 Apr 2026

Please see attached response letter

Attachment Submitted filename: Response to Reviewers.pdf

# Peer review: Decision Letter 1

**DOI:** 10.1371/journal.pone.0352872.r003

**Authors:** Weijun Yu, José Antonio Ortega

14 Jun 2026

PONE-D-25-63252R1

The Spatial Distribution of the U.S. Childfree Population: Does State Context Matter?

PLOS One

Dear Dr. Neal,

Thank you for submitting your manuscript to PLOS ONE. After careful consideration, we feel that it has merit but does not fully meet PLOS ONE’s publication criteria as it currently stands. Therefore, we invite you to submit a revised version of the manuscript that addresses the points raised during the review process.

The manuscript has been sent back to the reviewers, the academic editor was not available and I have taken over while relying on the same reviewers.

Reviewer 1 notes improvements in the manuscript but there are still some issues requiring clarification or change of language, like softening the language on the clustering or use of sample weights in all the analysis. If these changes are attended (or rebutted), the manuscrit could be accepted in the next revision without sending it back to the reviewers.

Please submit your revised manuscript by Jul 29 2026 11:59PM. If you will need more time than this to complete your revisions, please reply to this message or contact the journal office at plosone@plos.org. When you're ready to submit your revision, log on to https://www.editorialmanager.com/pone/ and select the 'Submissions Needing Revision' folder to locate your manuscript file.

Please include the following items when submitting your revised manuscript:

- A letter that responds to each point raised by the academic editor and reviewer(s). You should upload this letter as a separate file labeled 'Response to Reviewers'.
- A marked-up copy of your manuscript that highlights changes made to the original version. You should upload this as a separate file labeled 'Revised Manuscript with Track Changes'.
- An unmarked version of your revised paper without tracked changes. You should upload this as a separate file labeled 'Manuscript'.

If you would like to make changes to your financial disclosure, please include your updated statement in your cover letter. Guidelines for resubmitting your figure files are available below the reviewer comments at the end of this letter.

If applicable, we recommend that you deposit your laboratory protocols in protocols.io to enhance the reproducibility of your results. Protocols.io assigns your protocol its own identifier (DOI) so that it can be cited independently in the future. For instructions see: https://journals.plos.org/plosone/s/submission-guidelines#loc-laboratory-protocols. Additionally, PLOS ONE offers an option for publishing peer-reviewed Lab Protocol articles, which describe protocols hosted on protocols.io. Read more information on sharing protocols at https://plos.org/protocols?utm_medium=editorial-email&utm_source=authorletters&utm_campaign=protocols.

As the corresponding author, your ORCID iD is verified in the submission system and will appear in the published article. PLOS supports the use of ORCID, and we encourage all coauthors to register for an ORCID iD and use it as well. Please encourage your coauthors to verify their ORCID iD within the submission system before final acceptance, as unverified ORCID iDs will not appear in the published article. *Only*  the individual author can complete the verification step; PLOS staff *cannot*  verify ORCID iDs on behalf of authors.

We look forward to receiving your revised manuscript.

Kind regards,

José Antonio Ortega, Ph.D.

Academic Editor

PLOS One

Journal Requirements:

If the reviewer comments include a recommendation to cite specific previously published works, please review and evaluate these publications to determine whether they are relevant and should be cited. There is no requirement to cite these works unless the editor has indicated otherwise.

Please review your reference list to ensure that it is complete and correct. If you have cited papers that have been retracted, please include the rationale for doing so in the manuscript text, or remove these references and replace them with relevant current references. Any changes to the reference list should be mentioned in the rebuttal letter that accompanies your revised manuscript. If you need to cite a retracted article, indicate the article’s retracted status in the References list and also include a citation and full reference for the retraction notice.

[Note: HTML markup is below. Please do not edit.]

Reviewers' comments:

Reviewer's Responses to Questions

**Comments to the Author**

1. If the authors have adequately addressed your comments raised in a previous round of review and you feel that this manuscript is now acceptable for publication, you may indicate that here to bypass the “Comments to the Author” section, enter your conflict of interest statement in the “Confidential to Editor” section, and submit your "Accept" recommendation.

Reviewer #1: (No Response)

Reviewer #2: All comments have been addressed

**********

2. Is the manuscript technically sound, and do the data support the conclusions?

The manuscript must describe a technically sound piece of scientific research with data that supports the conclusions. Experiments must have been conducted rigorously, with appropriate controls, replication, and sample sizes. The conclusions must be drawn appropriately based on the data presented.

Reviewer #1: Yes

Reviewer #2: Yes

**********

3. Has the statistical analysis been performed appropriately and rigorously?

Reviewer #1: Yes

Reviewer #2: Yes

**********

4. Have the authors made all data underlying the findings in their manuscript fully available?

The PLOS Data policy requires authors to make all data underlying the findings described in their manuscript fully available without restriction, with rare exception (please refer to the Data Availability Statement in the manuscript PDF file). The data should be provided as part of the manuscript or its supporting information, or deposited to a public repository. For example, in addition to summary statistics, the data points behind means, medians and variance measures should be available. If there are restrictions on publicly sharing data—e.g. participant privacy or use of data from a third party—those must be specified.

Reviewer #1: Yes

Reviewer #2: Yes

**********

5. Is the manuscript presented in an intelligible fashion and written in standard English?

PLOS ONE does not copyedit accepted manuscripts, so the language in submitted articles must be clear, correct, and unambiguous. Any typographical or grammatical errors should be corrected at revision, so please note any specific errors here.

Reviewer #1: Yes

Reviewer #2: Yes

**********

6. Review Comments to the Author

Please use the space provided to explain your answers to the questions above. You may also include additional comments for the author, including concerns about dual publication, research ethics, or publication ethics. (Please upload your review as an attachment if it exceeds 20,000 characters)

Reviewer #1: Respected Authors:

The revised manuscript examines the spatial distribution of childfree adults in the United States using data from the CHIP50 survey (N = 20,118 respondents across all 50 states). The authors estimate state-level prevalence, assess spatial clustering using Moran’s I, and employ mixed-effects models to evaluate the relative influence of individual- and state-level predictors.

The revised version addresses several concerns raised in the initial review. Notably, the authors have:

• clarified the operationalization of childfree status, including full survey question wording

• improved the description of sampling and weighting procedures, specifying quota sampling and post-stratification weights

• added model specification details, including random intercepts and reporting of ICC values

• included a formal spatial clustering test (Moran’s I) with appropriate reporting

• incorporated a Data Availability Statement with repository information

• added an IRB statement confirming exemption

These revisions substantially improve the manuscript’s transparency and compliance with journal requirements. The study remains relevant and contributes novel insight into the geography of voluntary childlessness.

However, several issues remain that should be addressed prior to publication, primarily related to interpretation, methodological limitations, and reporting clarity.

The manuscript now meets many of the core requirements of PLOS ONE, including:

• originality of research

• appropriate analytical approach

• improved transparency and reproducibility

• compliance with ethics and data availability standards

Nevertheless, interpretive overreach and some remaining ambiguities limit the strength of the conclusions. These issues are addressable with moderate revision.

Major Comments

1. Interpretation of “No Spatial Clustering”

The revised manuscript appropriately includes a Moran’s I analysis and reports non-significant results (e.g., I = 0.108, p = 0.079) . However, the interpretation remains somewhat overstated.

The conclusion that the childfree population is “not clustered” should be further qualified the p-values are marginal (close to 0.05) and do not strongly support absence of spatial structure, and The analysis is limited to state-level adjacency, which may obscure clustering at smaller spatial scales.

Although the authors now clarify that clustering refers to “multi-state regions,” the manuscript should still emphasize that there is a failure to detect clustering different from the absence of spatial structure. Also, clustering may exist at sub-state levels (e.g., metropolitan areas)

2. Representativeness and Sampling Limitations

The revised manuscript improves the description of sampling by noting the quota sampling via online panels, and the post-stratification weights by demographic characteristics.

However, concerns remain regarding the data are still derived from a non-probability sample, which limits generalizability. The manuscript also states the data are “weighted to be representative,” but does not fully discuss limitations of this approach.

My recommendation here is that the authors should explicitly acknowledge the limitations of quota-based online samples, based on potential biases that weighting cannot fully correct, as they have implications for interpreting state-level differences

3. Use of Survey Weights in Multilevel Models

While weighting is clearly described for state-level prevalence estimation, it remains unclear whether survey weights were incorporated into the mixed-effects models.

Given that the weights are necessary for representativeness, and the multilevel models can be sensitive to weighting decisions, this omission is important.

My recommendation here is to clarify whether weights were used in the mixed-effects models, and if not, please provide justification

4. Model Interpretation and ICC

The authors report a very low intra-class correlation coefficient (ICC = 0.007) and conclude that state-level variation is negligible. While this is informative, the interpretation could be strengthened considering the following:

• A low ICC does not necessarily imply that state context is unimportant, particularly if relevant contextual variables are not fully captured.

• The near-zero variance may reflect measurement limitations at the state level, rather than substantive absence of contextual effects.

5. Selection of State-Level Variables

The authors provide additional justification for selected variables (e.g., abortion policy, education spending, cost of living) . However, the exclusion of variables such as political context or urbanization at the state level is justified on the basis that they are measured individually, but this does not fully address contextual vs compositional effects.

My recommendation here is to clarify more explicitly why certain contextual variables were excluded, and whether omitted variable bias may influence results.

Minor Comments

1. Clarity and Grammar

Methods section (data cleaning description). The sentence: “these data were further cleaned the data”

remains grammatically incorrect and should be revised .

Background section. The sentence: “we focus on individuals who are do not want to have children” contains a grammatical error and should be corrected .

2. Table and Figure Reporting: Table 1 now includes sample sizes, and standard errors, which is an improvement. However, the absence of confidence intervals may limit interpretability for some readers. While space constraints are understandable, the authors may consider including confidence intervals in supplementary materials.

3. Abstract: The abstract appropriately summarizes findings, but the statement that the childfree population is “not clustered” should be slightly softened to reflect statistical uncertainty.

Evaluation Against PLOS ONE Criteria

1. Originality: The manuscript presents original research addressing an understudied demographic phenomenon.

2. Technical Quality: The analyses are appropriate and improved in clarity; however, weighting and model specification require minor clarification.

3. Validity of Conclusions: Conclusions are broadly supported but require more cautious interpretation of spatial results.

4. Clarity of Presentation: The manuscript is generally clear, with minor grammatical issues remaining.

5. Ethical Standards: The manuscript now includes an appropriate IRB statement confirming exemption.

6. Data Availability: The manuscript complies with data transparency requirements, with data and code available via OSF.

Respected Authors:

The revised manuscript represents a substantial improvement and addresses most of the concerns raised in the initial review. The remaining issues primarily involve:

• clarification of weighting in multilevel models

• more cautious interpretation of spatial results

• minor textual corrections

These revisions are relatively straightforward and would strengthen the manuscript before publication.

Reviewer #2: The authors have made a number of meaningful revisions in response to the initial review. Overall, I find that my prior concerns have been adequately addressed.

The authors included more clarifying information on their selected variables, and elaborated on their explanation of the findings related to population size and urbanicity. They also included additional details about the CHIP50 project and addressed my concern related to Figure 3.

I found no remaining issues after reviewing the revisions. I therefore recommend the current manuscript for publication.

**********

7. PLOS authors have the option to publish the peer review history of their article (what does this mean?). If published, this will include your full peer review and any attached files.

If you choose “no”, your identity will remain anonymous but your review may still be made public.

**Do you want your identity to be public for this peer review?**  For information about this choice, including consent withdrawal, please see our Privacy Policy.

Reviewer #1: No

Reviewer #2: No

**********

[NOTE: If reviewer comments were submitted as an attachment file, they will be attached to this email and accessible via the submission site. Please log into your account, locate the manuscript record, and check for the action link "View Attachments". If this link does not appear, there are no attachment files.]

To ensure your figures meet our technical requirements, please review our figure guidelines: https://journals.plos.org/plosone/s/figures

You may also use PLOS’s free figure tool, NAAS, to help you prepare publication quality figures: https://journals.plos.org/plosone/s/figures#loc-tools-for-figure-preparation.

NAAS will assess whether your figures meet our technical requirements by comparing each figure against our figure specifications.

# Author comment: Author response to Decision Letter 2

**DOI:** 10.1371/journal.pone.0352872.r004

15 Jun 2026

Please see attached cover letter.

# Editor report: Decision Letter 2

**DOI:** 10.1371/journal.pone.0352872.r005

**Authors:** Weijun Yu, José Antonio Ortega, José Antonio Ortega

16 Jun 2026

The Spatial Distribution of the U.S. Childfree Population: Does State Context Matter?

PONE-D-25-63252R2

Dear Dr. Neal,

We’re pleased to inform you that your manuscript has been judged scientifically suitable for publication and will be formally accepted for publication once it meets all outstanding technical requirements.

Within one week, you’ll receive an e-mail detailing the required amendments. When these have been addressed, you’ll receive a formal acceptance letter and your manuscript will be scheduled for publication.

An invoice will be generated when your article is formally accepted. Please note, if your institution has a publishing partnership with PLOS and your article meets the relevant criteria, all or part of your publication costs will be covered. Please make sure your user information is up-to-date by logging into Editorial Manager at Editorial Manager® and clicking the ‘Update My Information' link at the top of the page. For questions related to billing, please contact billing support.

If your institution or institutions have a press office, please notify them about your upcoming paper to help maximize its impact. If they’ll be preparing press materials, please inform our press team as soon as possible -- no later than 48 hours after receiving the formal acceptance. Your manuscript will remain under strict press embargo until 2 pm Eastern Time on the date of publication. For more information, please contact onepress@plos.org.

Kind regards,

José Antonio Ortega, Ph.D.

Academic Editor

PLOS One

Additional Editor Comments (optional):

The minor changes pending from the previous revision have been addressed or clarified satisfactorily.

Reviewers' comments:

# Editor report: Acceptance letter

**DOI:** 10.1371/journal.pone.0352872.r006

**Authors:** Weijun Yu, José Antonio Ortega, José Antonio Ortega

PONE-D-25-63252R2

PLOS One

Dear Dr. Neal,

I'm pleased to inform you that your manuscript has been deemed suitable for publication in PLOS One. Congratulations! Your manuscript is now being handed over to our production team.

At this stage, our production department will prepare your paper for publication. This includes ensuring the following:

* All references, tables, and figures are properly cited

* All relevant supporting information is included in the manuscript submission,

* There are no issues that prevent the paper from being properly typeset

You will receive further instructions from the production team, including instructions on how to review your proof when it is ready. Please keep in mind that we are working through a large volume of accepted articles, so please give us a few days to review your paper and let you know the next and final steps.

Lastly, if your institution or institutions have a press office, please let them know about your upcoming paper now to help maximize its impact. If they'll be preparing press materials, please inform our press team within the next 48 hours. Your manuscript will remain under strict press embargo until 2 pm Eastern Time on the date of publication. For more information, please contact onepress@plos.org.

You will receive an invoice from PLOS for your publication fee after your manuscript has reached the completed accept phase. If you receive an email requesting payment before acceptance or for any other service, this may be a phishing scheme. Learn how to identify phishing emails and protect your accounts at https://explore.plos.org/phishing.

If we can help with anything else, please email us at customercare@plos.org.

Thank you for submitting your work to PLOS ONE and supporting open access.

Kind regards,

PLOS ONE Editorial Office Staff

on behalf of

Dr. José Antonio Ortega

Academic Editor

PLOS One
